# Evaluating the transstadial effects of *Bacillus velezensis* and pyriproxyfen, alone and in combination, on fitness-related traits of *Culex quinquefasciatus*

**DOI:** 10.1186/s13071-025-07039-9

**Published:** 2025-09-24

**Authors:** Abdullah A. Alomar, Barry W. Alto

**Affiliations:** 1 Infectious Disease Vector Research Laboratory, Department of Plant Protection, College of Food and Agricultural Sciences, King Saud University, 11451 Riyadh, Saudi Arabia; 2 Florida Medical Entomology Laboratory, Institute of Food and Agricultural Sciences, Department of Entomology and Nematology, University of Florida, Vero Beach, FL 32962 USA

**Keywords:** Survival, Natural bacterial agent, *Bacillus velezensis*, Mosquito control, Pyriproxyfen, Fecundity, Sublethal effects

## Abstract

**Background:**

Lethal and sublethal effects of exposure to chemical and microbial agents can alter many mosquito life history traits and provide opportunities for integrated mosquito control strategies to reduce the risk of disease transmission. The insect growth regulator pyriproxyfen (PPF) disrupts metamorphosis by mimicking juvenile hormone, which primarily targets mosquitoes during the pupal-adult transformation. Biological agents like *Bacillus velezensis* (*Bv*) show larvicidal activity against mosquitoes, which can work in concert with the mode of action of PPF to enhance overall mosquito population suppression.

**Methods:**

This study investigated how PPF and *Bv* alone or in combination impact *Culex quinquefasciatus* performance and population recruitment by assessing both lethal (adult emergence as a proxy for overall immature mortality) and sublethal effects on fitness-related traits (lifespan and reproductive outputs). Experimental bioassays were conducted under laboratory standard conditions to determine mortality, development duration, lifespan, size, and fecundity.

**Results:**

Both agents independently reduced adult mosquito emergence, with the combination treatment producing the greatest overall reduction. When applied together, PPF and *Bv* significantly shortened adult female lifespan and reduced fecundity and hatching success of the offspring compared to individual treatments and the control. The combined treatment produced the most pronounced reductions across these life-history traits, indicating an additive effect.

**Conclusions:**

These findings highlight the potential of integrating PPF with a natural bacterial biocontrol agent through strong lethal and sublethal effects across multiple life stages of *Cx. quinquefasciatus*, including reduced adult lifespan and reproduction. Such an integrated approach can enhance the effectiveness of vector control while providing a sustainable and promising strategy to lower the risk of mosquito-borne disease transmission.

**Graphical abstract:**

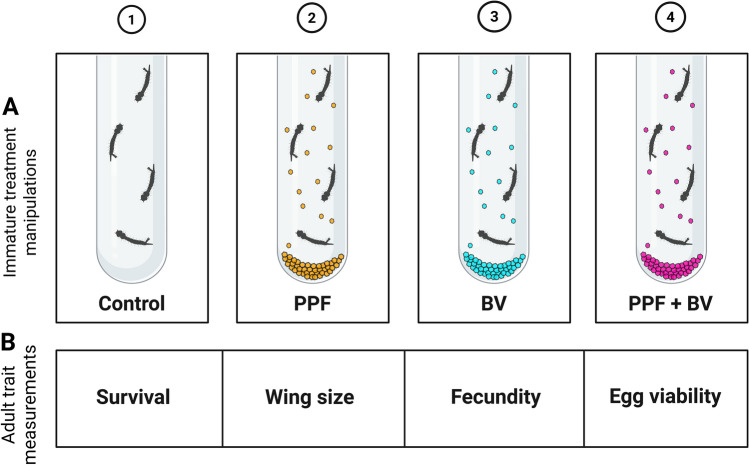

## Background

Control of mosquito vectors remains a critical public health challenge due to their role in transmitting of infectious diseases, such as dengue, Mayaro, and West Nile. Among these vectors, *Culex quinquefasciatus* is of particular concern due to its widespread distribution and its role in transmitting human and animal pathogens like West Nile virus and lymphatic filariasis, a parasitic disease affecting millions worldwide [1]. Its wide distribution, adaptability to urban environments, and role in disease transmission make it a key target for control efforts. Although chemical insecticides remain widely used in mosquito control, especially in endemic regions, growing concerns about insecticide resistance and negative environmental impacts have prompted a shift toward integrated vector management strategies that combine multiple control agents, including biological and environmental approaches, to achieve effective and sustainable mosquito control. While the combined use of insect growth regulators (e.g., pyriproxyfen) and bacterial biocontrol agents offers a promising alternative to traditional insecticides, repeated exposure may exert selective pressures that could lead to resistance development in mosquito populations. Therefore, evaluating the long-term sustainability and potential evolutionary outcomes of such integrated strategies remains an important consideration for vector management [1].

Among emerging biological tools, *Bacillus velezensis* (*Bv*) has gained attention because of its mosquitocidal properties. This Gram-positive, spore-forming bacterium produces a range of bioactive metabolites with antimicrobial and insecticidal activity [2–4]. Recent studies demonstrate its potent larvicidal effects against several mosquito species, supporting its potential as an effective biological control agent [4–6]. Furthermore, its highly selective mode of action ensures minimal impact on non-target organisms, making it a favorable candidate for mosquito control while minimizing environmental impact [7].

Insect growth regulators, such as pyriproxyfen (PPF), represent another important tool in mosquito management [8]. Pyriproxyfen is increasingly used in mosquito control programs because of its efficacy at low application rates and generally low toxicity to non-target organisms. However, some aquatic invertebrates may be susceptible under conditions of environmental accumulation or misapplication, and concerns about persistence and resistance development are emerging and warrant consideration [8]. Pyriproxyfen acts at the pupal stage by disrupting the transition to adulthood, thereby preventing adult emergence [9]. Because it does not induce mortality during the larval stages, its efficacy can be enhanced by incorporating other larval mortality factors into the control strategy, such as microbial larvicides or natural predators [10, 11].

Combining PPF with *Bv* presents an opportunity to leverage the distinct modes of action of each agent. *Bacillus velezensis* can reduce larval density through early-stage mortality, while PPF targets surviving pupae. This assumption predicts that PPF and *Bv* act in combination to inhibit the recruitment of adults and limits the associated probability of pathogen transmission. In addition to reducing adult emergence, such combinations may influence important fitness-related traits, including development duration, body size, longevity, and fecundity. These effects can further influence mosquito population dynamics and transmission of disease-causing pathogens. Furthermore, the use of a biological control agent such as *Bv* in combination with PPF provides an alternative mosquito management strategy that can help reduce reliance on traditional insecticides.

This study investigates the individual and combined effects of PPF and *Bv* on *Cx. quinquefasciatus*, a widespread vector of public health importance. Specifically, we assess their impact on development duration of immatures, adult emergence, and phenotypic traits of surviving mosquitoes that are potentially relevant to vectorial capacity, such as adult longevity, body size, and fecundity. Understanding the combined effects of these two control agents will contribute to the development of more effective, sustainable approaches to mosquito population management.

## Methods

### Mosquito rearing

*Culex quinquefasciatus* mosquitoes used in this study were originally collected as larvae from a small pool in Riyadh city during 2021 and were maintained under standard insectary conditions prior to this study. Mosquito egg rafts were collected and placed in larval rearing trays containing 1.5 l water. Upon hatching, larvae in each tray were provided with 0.2 g of TetraMin^®^ fish food daily as the larval diet. The rearing environment was maintained at 27 ± 1 °C, 60 ± 10% relative humidity, and a 12:12 h light–dark photoperiod. Pupae were collected daily using a Pasteur pipette and transferred to small plastic cups containing water. These cups were placed in adult rearing cages (30 × 30 × 30 cm, BugDorm, BioQuip Products, California, USA). Newly emerged adults were fed 10% sucrose solution ad libitum via cotton pads, which were replaced daily to prevent desiccation and microbial growth. Adult females were offered blood meals twice per week using a Hemotek membrane feeding system (Hemotek Ltd., Great Harwood, UK) with defibrinated  sheep blood (Saudi Prepared Media Laboratory Company Ltd., Riyadh, Saudi Arabia). The feeder was made available for a 1-h period. Following blood feeding, an oviposition cup with dechlorinated water was placed inside the cages for a 120-h period to allow females to lay eggs. Collected egg rafts were used to maintain colony propagation and initiate experimental cohorts.

### Bacterial strain isolation and crude toxin preparation

The bacterial strain *Bv*-WHk23 used in this study was originally isolated from soil collected from Wadi Hanifah, Riyadh, as described previously [5]. The bacterial strain was cultured in 300 ml NB2 medium in a sterile 1-l flask and incubated at 30 °C for 48 h with continuous shaking at 200 rpm. After incubation, the bacterial culture was centrifuged at 13,000 × *g* for 10 min at 4 °C, and the supernatant was collected. To precipitate the crude toxin, the supernatant was acidified to pH 2 using 6N HCl and stored at 4 °C overnight. The precipitate was recovered by centrifugation at 9000 × *g* for 30 min at 4 °C, suspended in dechlorinated water, and neutralized to pH 7 with 1 N NaOH. The resulting crude toxin was lyophilized, weighed, and stored at 4 °C until use in subsequent experiments.

### Mosquito responses to *Bacillus velezensis* and pyriproxyfen

Dose-response bioassays were conducted to estimate sublethal concentrations of *Bv* crude toxin and PPF for subsequent experiments. A *Bv* stock solution (1 mg/ml) and PPF stock solution (1 µg/ml) were prepared separately in dechlorinated water and serially diluted. The test concentrations ranged from 0 to 100 µg/ml for *Bv* and from 0 to 0.000095 µg/ml for PPF. For each concentration, 20 third-instar *Cx. quinquefasciatus* larvae were placed in plastic cups containing 200 ml treated water. Each concentration, including untreated controls, was replicated three times with a total of 60 larvae per treatment. Larvae were maintained at 27 ± 1 °C with a 12:12 h light-dark cycle. For *Bv*, larval mortality was assessed after 24 h of exposure, and larvae unresponsive to probing were recorded as dead. For PPF, larvae were exposed for 24 h and then transferred to clean dechlorinated water, where they were reared until adult emergence. PPF-induced mortality was recorded as the failure to emerge successfully into adults, reflecting its action during pupation. Bioassays were repeated if control mortality exceeded 10%. Low concentrations (e.g., LC₃₀) were estimated using probit regression analysis. The LC₃₀ for each compound was used in subsequent experiments to evaluate transstadial and sublethal effects on immature development duration and adult life-history traits.

### Immature treatment manipulation and mosquito trait assessment

Three hundred first-instar *Cx. quinquefasciatus* larvae were allocated to experimental trays (each serving as an independent replicate) containing 1.5 l dechlorinated water and 0.2 g TetraMin fish food. Larvae were randomly assigned to one of four treatment groups: Control (untreated), PPF at 0.00002 µg/ml, *Bv* at 17.92 µg/ml, and PPF + *Bv* (combined treatment at the same concentrations). Each treatment group was replicated three times. When larvae reached the third instar, the designated concentrations of PPF and/or *Bv* were added to the appropriate trays. Larvae were exposed to the treatments for 24 h, after which they were transferred to clean dechlorinated water to complete development. For the combined PPF + *Bv* group, both agents were applied simultaneously, and larvae were similarly transferred to clean water after 24 h. This 24-h exposure period was selected to simulate realistic, time-limited contact with control agents in natural environments, where concentrations may decline rapidly because of environmental factors such as sunlight, thus supporting the ecological relevance of short-duration exposure assays. All experimental trays were monitored daily, and newly formed pupae were transferred to individual water-filled vials with lids to allow adult emergence. For each replicate, developmental duration (from hatching to pupation, pooled across sexes) was recorded in days. Adult emergence was also recorded by sex (males and females) to assess overall developmental success.

### Mosquito longevity

To evaluate the impact of larval treatments on adult female longevity, a total of 150 newly emerged females per treatment replicate (i.e., 50 individuals from each rearing tray) were monitored for daily survival. Females were housed in 16-ounce cardboard cages under standard insectary conditions (27 ± 1 °C, 60 ± 10% RH, 12:12 h light-dark cycle) and provided continuous access to 10% sucrose solution via soaked cotton pads, which were replaced daily. Mortality was recorded daily throughout the duration of the experiment.

### Fecundity, egg viability, and wing length

Following adult emergence, mosquitoes were held in mixed-sex cages for 5–8 days to allow natural mating under standard insectary conditions (27 ± 1°C, 60 ± 10% RH, 12:12 h light–dark cycle) with continuous access to 10% sucrose solution via cotton pads. Prior to blood feeding, females were deprived of sucrose overnight. Mosquitoes were then offered a single defibrinated sheep blood meal for 1 h using a Hemotek membrane feeding system preheated to 37 °C. Approximately 3–4 h after the blood meal, 30 fully engorged females were randomly selected per treatment group for assessment of fecundity, fertility, and wing length. Females were briefly immobilized using cold treatment (3–5 min) to facilitate individual transfer into oviposition cages. Each female was housed individually in a cylindrical oviposition cage (16 oz cup) fitted with mesh covering and provided with a 10% sucrose solution (via cotton pad) and a 100-ml oviposition cup filled with dechlorinated water. The oviposition cup was left in the cage for 5 days post-blood feeding to allow for egg laying. Mosquitoes were monitored daily, and the total number of eggs laid per female was recorded as a measure of fecundity. Egg viability (fertility) was assessed approximately 72 h after oviposition by counting the number of larvae (neonates) hatched per egg raft. Adult female body size was estimated by measuring wing length, used as a proxy, from the same 30 females per treatment group used in the fecundity assay [12, 13]. A single wing (typically the right wing) was dissected from each female after oviposition, mounted on a glass slide using double-sided tape, and measured under a dissecting microscope equipped with an eyepiece micrometer. Wing length was measured in millimeters from the alular notch to the distal margin, excluding the fringe.

## Statistical analysis

Lethal concentrations (e.g., LC₃₀) for *Bv* and PPF were estimated using probit regression based on preliminary dose-response bioassays. For the main experiment, treatment effects on mosquito traits, including time to pupation, adult emergence, wing length, and fecundity, were assessed using one-way analysis of variance (ANOVA). Prior to analysis, assumptions of normality and homogeneity of variance were verified. Data normality was assessed using the Shapiro-Wilk test, which confirmed that all datasets met the assumption of normal distribution. Additional tests (D’Agostino & Pearson, Anderson-Darling, and Kolmogorov-Smirnov) were also consistent with this result. Post hoc comparisons among treatment groups were conducted using Tukey’s Honestly Significant Difference (HSD) test to identify pairwise differences. Adult female survival was analyzed using Cox proportional hazards regression to evaluate treatment effects on longevity (data censored after 40 days). Individuals derived from replicate rearing trays were treated as independent experimental units. All statistical analyses were performed using SAS statistical software version 9.4 (SAS Institute Inc., Cary, NC) and GraphPad Prism version 10 (GraphPad Software, La Jolla, CA, USA), with statistical significance set at *p* < 0.05.

## Results

### Immature development time and adult emergence

**Fig. 1:**
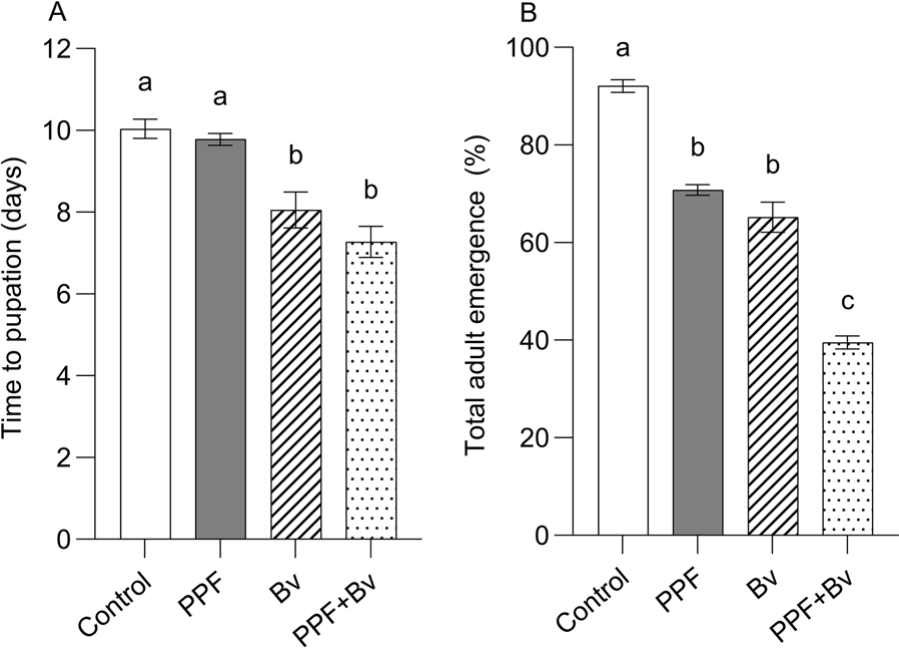
Treatment effects on pre-adult mosquito traits. **A** Time to pupation (hatch to pupation) in days. **B** Total adult emergence. Bars represent means ± standard error of the means. Different letters indicate statistically significant differences between treatment groups, after correcting for multiple comparisons (*p* < 0.05; Tukey’s HSD)

Treatment significantly affected the mean time to pupation (F_3, 8_ = 17.19, *p* = 0.0008). Larvae in the control group had the longest development duration (10.0 ± 0.26 days), followed by the PPF group (9.8 ± 0.17 days). In contrast, larvae exposed to *Bv* (8.1 ± 0.48 days) and the PPF + *Bv* combination (7.2 ± 0.41 days) pupated significantly faster than those in the control or PPF groups (Fig. 1A). There was no significant difference between control and PPF treatments (*p* = 0.94), whereas both *Bv* and PPF + *Bv* significantly shortened immature development duration relative to the control (*p* < 0.01).

Adult emergence, used as a proxy for cumulative immature (larval and pupal) mortality, was also significantly influenced by treatment (F₃, ₈ = 133.0, *p* < 0.0001). The highest emergence rate (i.e. lowest mortality) occurred in the control group (91.4 ± 0.9%), followed by PPF (70.8 ± 1.1%) and *Bv* (70.7 ± 0.8%). The PPF + *Bv* group exhibited the lowest emergence (40.3 ± 0.6%) (Fig. 1B), indicating a strong additive effect of the combined treatment. Both PPF and *Bv* alone resulted in significantly lower emergence than the control (*p* < 0.01), and the combination treatment significantly reduced emergence compared to either agent alone (*p* < 0.001). Sex ratio (males: females) was approximately balanced across treatments, with mean values ranging from 1.07 to 1.15, indicating no strong sex-specific differences in emergence among treatment groups.

### Adult female longevity

**Fig. 2:**
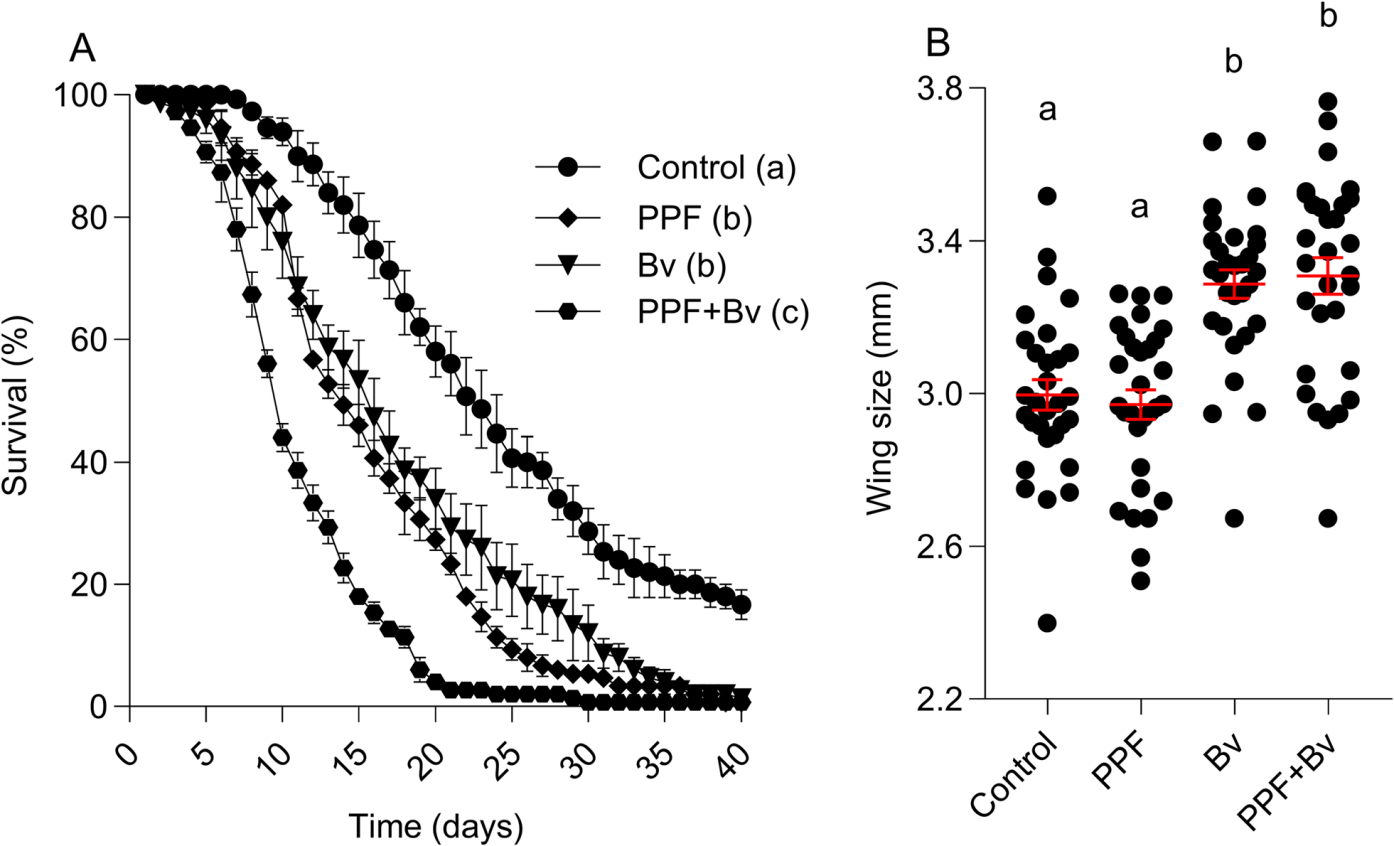
Treatment effects on lifespan and size of adult mosquitoes. **A** Female survival curves. **B** Wing length. Data shown represent the means ± standard error of the means. Different letters indicate statistically significant differences between treatment groups, after correcting for multiple comparisons (*p* < 0.05; Tukey’s HSD)

Treatment significantly affected adult female longevity (Cox proportional hazards regression, log-likelihood test: *χ*^2^ = 168.2, df 3, *p* < 0.0001). Relative to the control group, females from the *Bv* (HR 1.86, 95% CI 1.46–2.37), PPF (HR 2.51, 95% CI 1.97–3.20), and PPF + *Bv* (HR 5.29, 95% CI 4.11–6.82) treatments had significantly higher mortality risks. The control group exhibited the longest lifespan, with several individuals surviving beyond day 40, while those exposed to the combined PPF + *Bv* treatment experienced the shortest lifespan. The PPF + *Bv* group showed the steepest decline in the survivor function, with < 10% of females remaining by day 19 and complete mortality occurring by day 29, indicating additive detrimental effects on adult longevity from combined larval exposure (Fig. 2A).

### Adult female body size (wing length)

Length was significantly influenced by larval treatment (F_3, 116_ = 19.53, *p* < 0.0001). The largest females emerged from the PPF + *Bv* group (3.41 ± 0.04 mm), followed by the *Bv* group (3.35 ± 0.03 mm). Control females were smaller (2.99 ± 0.03 mm), and the smallest wing lengths were observed in the PPF group (2.97 ± 0.04 mm) (Fig. 2B). These results suggest that microbial and chemical exposures during immature development can alter adult size and growth.

### Adult female fecundity and egg viability

**Fig. 3:**
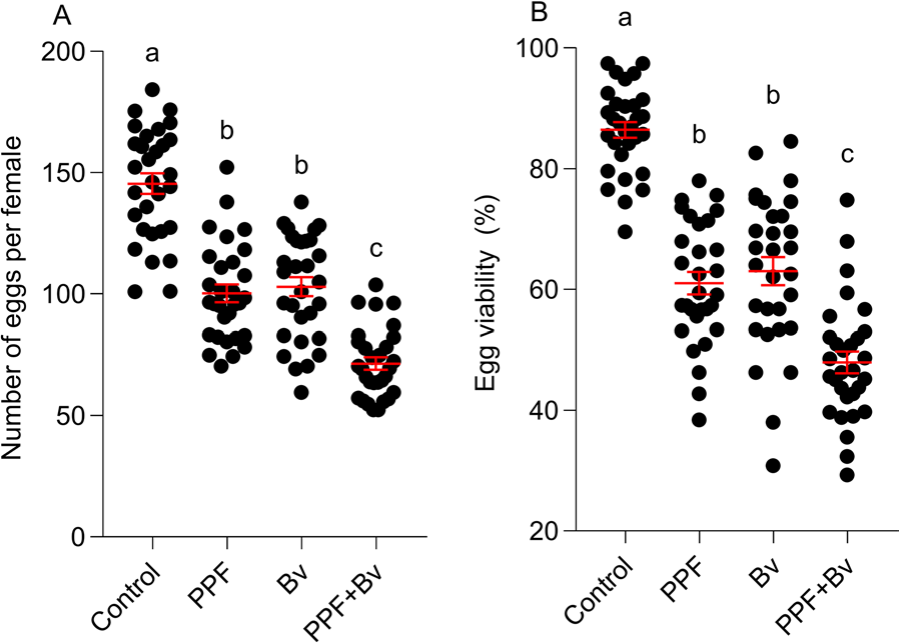
Treatment effects on reproductive output of female mosquitoes. **A** Fecundity (number of laid eggs per female). **B** Egg viability (percentage of hatched larvae per female). Horizontal lines indicate means ± standard error of the means. Different letters indicate statistically significant differences between treatment groups, after correcting for multiple comparisons (*p* < 0.05; Tukey’s HSD)

Fecundity (number of eggs laid per female) varied significantly among treatments (F_3, 116_ = 69.83, *p* < 0.0001). Control females laid the most eggs (154.03 ± 4.1), while lower values were recorded for the *Bv* (102.1 ± 3.9), PPF (100.2 ± 3.6), and PPF + *Bv* (71.5 ± 2.5) groups. The PPF + *Bv* treatment led to the greatest reduction in egg production (Fig. 3A).

Egg viability, measured as percent hatch, was also significantly affected (F_3, 116_ = 74.36, *p* < 0.0001). The highest hatch rate was recorded in the control group (86.3 ± 1.3%), followed by *Bv* (65.5 ± 2.3%) and PPF (61.0 ± 2.2%), while the lowest hatchability was observed in the PPF + *Bv* group (48.0 ± 2.4%), indicating a strong negative effect on fertility from combined larval exposure (Fig. 3B).

To better estimate reproductive success, we calculated total reproductive output (TRO) per female by multiplying mean fecundity by egg hatch rate. TRO was highest in the control group (132.9 ± 5.0 viable larvae/female), followed by *Bv* (66.8 ± 3.4), PPF (61.2 ± 3.3), and PPF + *Bv* (34.4 ± 1.9). These results indicate that combined larval exposure to PPF and *Bv* led to the greatest reduction in reproductive success, further highlighting the additive impact of dual treatment on adult mosquito fitness Table 1.

**Table 1:** Mean fecundity (number of eggs laid), egg viability (% hatching), and total reproductive output (product of fecundity and the proportion of eggs that successfully hatched, i.e., egg viability)

Treatment	Fecundity	SE	Egg viability	SE	Total reproductive output
Control	154.03	4.1	86.3	1.3	132.9
*Bv*	102.1	3.9	65.5	2.3	66.87
PPF	100.2	3.6	61	2.2	61.12
PPF + *Bv*	71.5	2.5	48	2.4	34.32

## Discussion

In this study, we assessed multiple life history traits of *Cx. quinquefasciatus* following larval exposure to PPF, *Bv*, and their combination. Treatments with either PPF or *Bv* significantly reduced adult emergence, and the combination (PPF + *Bv*) yielded the lowest emergence rates, indicating an additive suppression effect. Adult lifespan was also shortened by both agents, with the most pronounced reduction observed in the combined treatment group. Beyond survival outcomes, we observed transstadial effects on adult traits: females from the *Bv* and PPF + *Bv* treatments exhibited larger body sizes, yet this did not translate into improved reproductive performance. In fact, fecundity and egg viability were significantly reduced in all treatment groups, with the greatest reductions observed under combined exposure. These results suggest that larval exposure to microbial and chemical agents can negatively affect adult fitness, thereby compounding their impact on mosquito population dynamics and supporting the potential use of these agents in integrated control strategies. Moreover, the observed reduction in lifespan, attributable to sublethal effects, is likely to impede their ability to serve as vectors of pathogens affecting human health.

Unlike conventional larvicides that directly kill larvae, PPF allows larvae to develop to the pupal stage but prevents successful metamorphosis into adults [9]. This mode of action allows other biotic factors (e.g., larval competition for nutrient resources) and larvicides (e.g., microbial larvicides) that contribute to larval mortality to take their toll before PPF-induced pupal mortality occurs. This approach aligns with established knowledge on the timing of density dependence and its influence on mosquito immature mortality and population dynamics [14, 15]. Our findings on developmental duration and adult size (wing length) suggest that the delayed action of the PPF offers distinct advantages for mosquito control. Specifically, the control and PPF treatments were associated with the longest development times and smallest adults, attributes predicted to incur fitness costs. Moreover, the PPF treatment significantly lowered adult emergence and impaired female longevity, further highlighting its potential to suppress mosquito populations and impede their ability to transmit pathogens. Results showed strong suppression effects of *Bv* and PPF on adult emergence, with *Bv* exhibiting high larvicidal activity and PPF interfering with normal metamorphosis. Combination treatments produced additive effects, supporting the potential for integrating microbial and PPF control agents into vector management programs. These results are consistent with previous studies showing greater suppression of mosquito populations when a PPF was combined with biopesticides or biological control agents. For instance, evaluation of PPF and spinosad (targets nicotinic acetylcholine receptors of the nervous system) to control *Aedes aegypti* showed that the PPF inhibited adult emergence, whereas spinosad caused larval mortality, providing a broader and improved control strategy compared with either agent alone [10]. Similarly, studies have shown that the combination of PPF with a dipteran predator significantly reduced adult mosquito emergence rates compared with either agent alone [11, 16]. Together, these results suggest that integrating PPF with other sources of larval mortality, such as biological control agents, may enhance mosquito suppression and improve vector control strategies. However, demographic responses of mosquitoes to extrinsic sources of mortality, including the timing of treatment application, can differ among mosquito species [17]. Therefore, it is not entirely clear whether the observed findings in *Cx. quinquefasciatus* follow similar patterns in other mosquito species.

Exposure to *Bv* significantly affected phenotypic traits of *Cx. quinquefasciatus*, including immature developmental duration and fecundity. Although PPF did not affect immature development, exposure to *Bv* significantly accelerated larval development and led to the emergence of larger adult mosquitoes. *Bacillus velezensis*-exposed mosquitoes developed faster and emerged as adults earlier than those in the control groups. This rapid development may be attributed to density-mediated effects, where mortality among immature stages due to *Bv* exposure reduced competition for resources, allowing survivors to develop faster and achieve larger body sizes. Similar effects have been observed in other studies, where exposure to insecticides during larval stages shortened development time and increased adult body size in mosquito survivors that experienced a sublethal effect [18–20]. Despite exhibiting larger body size, *Bv*- and PPF + *Bv*-exposed females did not exhibit a size-associated reproductive advantage, where larger mosquitoes have higher fecundity than smaller conspecifics. Rather, they produced fewer eggs with lower hatch rates compared to control females. This result contradicts the commonly observed positive correlation between body size and fecundity in mosquitoes. Although the effects of *Bv* on reproduction have not been previously studied, similar patterns have been reported for other bacterial larvicides. For example, *Anopheles coluzzii* exposed to *Bacillus thuringiensis israelensis* (*Bti*) developed longer wings but laid fewer eggs at higher exposure levels [21]. Likewise, both *Aedes albopictus* and *Ae. aegypti* exposed to *Bti* showed reduced fecundity [22, 23]. Exposure to *Bacillus sphaericus* has also been associated with declines in female egg production and longevity of adults (females and males) in *Cx. quinquefasciatus* and *Ae. aegypti* [24]. These findings suggest that certain bacterial exposures during larval development can induce long-lasting physiological changes that impair adult fitness. Mosquito exposure to select bacterial agents may specifically affect fecundity, which may or may not be associated with other traits, although the mechanism is not entirely clear. Potential mechanisms may involve altered energy allocation, immune activation, or disruptions to gut microbiota, as previously observed in *Bti*-tolerant *Ae. albopictus* [23]. More recently, a transcriptome analysis showed that the bacterial agent *Lysinibacillus sphaericus* (previously *Bacillus sphaericus*) inhibits fecundity in *An. dirus* by downregulating vitellogenin expression via inhibition of lysosomal function and the Akt/TOR (target of rapamycin) signaling pathway in adult mosquitoes [25].

In addition to *Bacillus* control agents influencing the fecundity of mosquitoes, other chemical larvicides can have similar outcomes on reproductive biology. *Culex quinquefaciatus* larvae exposed as fourth instars to malathion or methoprene at an LC_50_ level resulted in adult females that produced fewer eggs in the first gonotrophic cycle (40–50% reduction) than control mosquitoes (unexposed) [26]. Similarly, a 30–40% reduction in egg production was observed in the second gonotrophic cycle for these same insecticide-exposed mosquitoes relative to controls. Also, a reduction in hatching rate was observed for *Cx. quinquefasciatus* following exposure to methoprene (LC_50_) but not malathion [26]. Methoprene is an insect growth regulator (IGR) and juvenile hormone analog, whereas malathion is an organophosphate, which acts as an acetylcholinesterase inhibitor essential for normal nerve function. Surprisingly, similar results were not observed for *Cx. quinquefasciatus* exposed to propoxur (carbamate) and resmethrin (pyrethroid). Propoxur is an acetylcholinesterase inhibitor. Resmethrin acts by preventing the closure of the voltage-gated sodium channels in the axonal membranes. These results suggest that sublethal exposure to larvicides during development may impose physiological costs that extend into adulthood and constrain reproduction. Furthermore, different classes of insecticides may vary in the degree to which they impose physiological costs on mosquito reproduction.

Similar to the effects observed with *Bv*, exposure to PPF during immature development altered female lifespan and fecundity, leading to transstadial effects that shortened adult lifespan and decreased reproductive output in the surviving mosquitoes. These observations are consistent with previous studies that showed exposure to PPF during the larval stage induced transstadial effects that led to shortened adult lifespan in surviving *Ae. aegypti* mosquitoes [11, 16]. These effects were observed even at low concentrations, affecting the overall lifespan of adults [11, 16]. Taken together, the combination of PPF and *Bv* in this study resulted in significantly greater reductions in lifespan and fecundity than either treatment alone, highlighting its potential to enhance mosquito population suppression. The consistent reductions in adult longevity, body size, and reproductive output observed across treatments suggest coherent transstadial effects of larval exposure on adult stages. These outcomes emphasize how larval-stage interventions can extend their impact into adulthood, reducing mosquito vectorial capacity primarily through shortened female lifespan and reinforcing the value of integrated approaches targeting multiple life history traits [27].

While these laboratory findings are promising, they may not fully reflect the complexities of natural environments. Factors such as water quality, sunlight exposure, and microbial degradation can influence the persistence and efficacy of these agents under field conditions. Also, our use of a single cohort of mosquitoes, targeting exposure during the third instar, does not reflect the reality of multiple and overlapping cohorts of mosquitoes (e.g., intra- and interspecific interactions) that occupy container habitats, which requires further investigation. Furthermore, long-term and repeated use of combined treatments could impose selection pressures that contribute to the development of resistance. Although resistance to *Bv* has not yet been documented in mosquito populations, integrating it with other control agents that have different models of action could help mitigate future risks and preserve long-term efficacy. Lastly, the environmental safety of deployment of bacterial and IGR control agents broadly in the field must be considered, particularly for non-target invertebrates, which remain understudied in ecotoxicological assessments. These considerations highlight the need for future research to evaluate the ecological relevance, sustainability, and operational feasibility of such integrated control strategies in semi-field and field environments.

## Conclusions

This study demonstrates that PPF and bacterial agents, both individually and in combination, significantly impact the life history traits of *Cx. quinquefasciatus*, including immature development, adult emergence, adult survival, and reproduction. The larvicidal activity of *Bv* complements the pupal-targeting action of PPF, resulting in enhanced suppression of adult emergence when applied together. Importantly, we observed transstadial effects of larval exposure on adult fitness, with combined treatments leading to reduced adult longevity and lower fecundity and fertility. These findings suggest possible physiological trade-offs associated with larvicide exposure. These findings underscore the potential of combining PPF and *Bv* for enhanced mosquito population suppression, supporting their integration into vector management programs as a sustainable strategy for controlling of emerging vector-borne diseases. Collectively, our results support the integration of *Bv* and PPF as an effective strategy for vector control, with implications for reducing mosquito vectorial capacity and the risk of vector-borne pathogen transmission.

## Data Availability

All relevant data generated or analyzed during this study are included in this published article.

## Abbreviations

IGR: Insect growth regulator
PPF: Pyriproxyfen
Bv: *Bacillus velezensis*
LC₅₀: Lethal concentration 50%
ANOVA: Analysis of variance
Oz: Ounce
NB: Nutrient broth
pH: Potential of hydrogen
N HCl: Normal hydrochloric acid
NaOH: Sodium hydroxide
